# Anti-Diabetic Countermeasures Against Tobacco Smoke-Dependent Cerebrovascular Toxicity: Use and Effect of Rosiglitazone

**DOI:** 10.3390/ijms20174225

**Published:** 2019-08-29

**Authors:** Farzane Sivandzade, Luca Cucullo

**Affiliations:** 1 Department of Pharmaceutical Sciences, Texas Tech University Health Sciences Center, Amarillo, TX 79106, USA; 2 Center for Blood-Brain Barrier Research, Texas Tech University Health Sciences Center, Amarillo, TX 79106, USA

**Keywords:** rosiglitazone, repurposing, oxidative stress, blood-brain barrier, tight junctions, Nrf2, PPARγ, brain, disease, cerebrovascular

## Abstract

Tobacco smoking (TS) is one of the most addictive habit sand a main public health hazards, impacting the vascular endothelium through oxidative stress (OS) stimuli, exposure to nicotine, and smoking-induced inflammation in a dose-dependent manner. Increasing evidence also suggested that TS increases glucose intolerance and the risk factor of developing type-2 diabetes mellitus (2DM), which, along with TS, is connected to blood–brain barrier (BBB) injuries, and heightens the risk of cerebrovascular disorders. Although the exact mechanism of rosiglitazone (RSG) is unknown, our previous in vitro work showed how RSG, an oral anti-diabetic drug belonging to the family of thiazolidinedione class, can protect BBB integrity through enhancement of nuclear factor erythroid 2-related factor (Nrf2) activity. Herein, we have validated the protective role of rosiglitazone against TS-induced BBB impairment in vivo. Our results revealed that RSG as a peroxisome proliferator-activated receptor gamma (PPARγ), activates counteractive mechanisms primarily associated with the upregulation of Nrf2 and PPARγ pathways which reduce TS-dependent toxicity at the cerebrovascular level. In line with these findings, our results show that RSG reduces inflammation and protects BBB integrity. In conclusion, RSG offers a novel and promising therapeutic application to reduce TS-induced cerebrovascular dysfunction through activation of the PPARγ-dependent and/or PPARγ-independent Nrf2 pathway.

## 1. Introduction

A vast number of deaths worldwide, are attributed to smoking, as a consequence of its effects on the vascular system in the body [1,2]. As a major component of the vascular system, the endothelial cells are significantly impaired as a result of exposure to the toxic chemicals, free radicals, aromatic compounds and nicotine contained within tobacco smoke (TS). Endothelial function is critical to maintain the integrity, homeostasis and detoxifying role of the blood–brain barrier (BBB) [1,3,4,5,6,7]. The exact components of cigarette smoke and the mechanism of the pathophysiological link between smoking and vascular injury are not fully specified. The mechanism of vascular damage induced by cigarette smoking is multifaceted; dysfunction of the BBB through activation of oxidative, inflammatory and immune responses leads to pathogenesis and progression of cerebrovascular and neurodegenerative disorders, including stroke, Alzheimer’s disease (AD), Parkinson disease (PD), amyotrophic lateral sclerosis (ALS), depression, vascular dementia and Huntington’s disease (HD) [1,2,5,8,9,10,11,12,13]. In fact, the selectivity of the BBB, a dynamic and complex interface between the blood and the central nervous system (CNS), allows some nutrients to transport between the peripheral circulation and the brain, while it prevents many toxic compounds and pathogens from entering the brain [14,15,16,17]. There is now a wealth of evidence suggesting the major role of oxidative stress in endothelial dysfunction in the cerebrovascular level [18]. Despite the valid evidence for the significant link between cigarette smoking and vascular impairment, the impact of TS exposure on the BBB has not been completely addressed [1,13,19]. In the recent work of our group, the involvement of common pathogenic modulators of BBB impairment was confirmed so that chronic cigarette smoking and hyperglycemia (HG) carried similar risks for cerebrovascular diseases and stroke, sharing similar pathogenic mechanisms [20,21,22]. This result accounts for the reason for the possible application of anti-diabetic drugs to prevent/reduce BBB damage promoted by the chronic TS exposure. Rosiglitazone (RSG) is a member of the thiazolidinedione family of antidiabetic agents that can improve insulin sensitivity through modulating adiponectin gene expression in muscle and adipose tissue, and inhibits hepatic gluconeogenesis. RSG is also considered as a potent and selective transcription factor peroxisome proliferator-activated receptor (PPARγ) agonist which is a nuclear receptor that regulates numerous genes implicated in glucose homeostasis, and fatty acid metabolism [23,24,25]. In humans, PPAR receptors are found in key target tissues for insulin action, such as adipose tissue, skeletal muscle, and liver. Despite the unknown mechanism of RSG, numerous studies and our previous work has confirmed the protective effect of RSG against oxidative damage [2,23,24,26,27]. The aim of the present study is to validate and assess the previous results using animal models in vivo and to confirm RSG’s role in the activation of counteractive antioxidative mechanisms to reduce TS toxicity at the BBB.

## 2. Result

**Figure 1:**
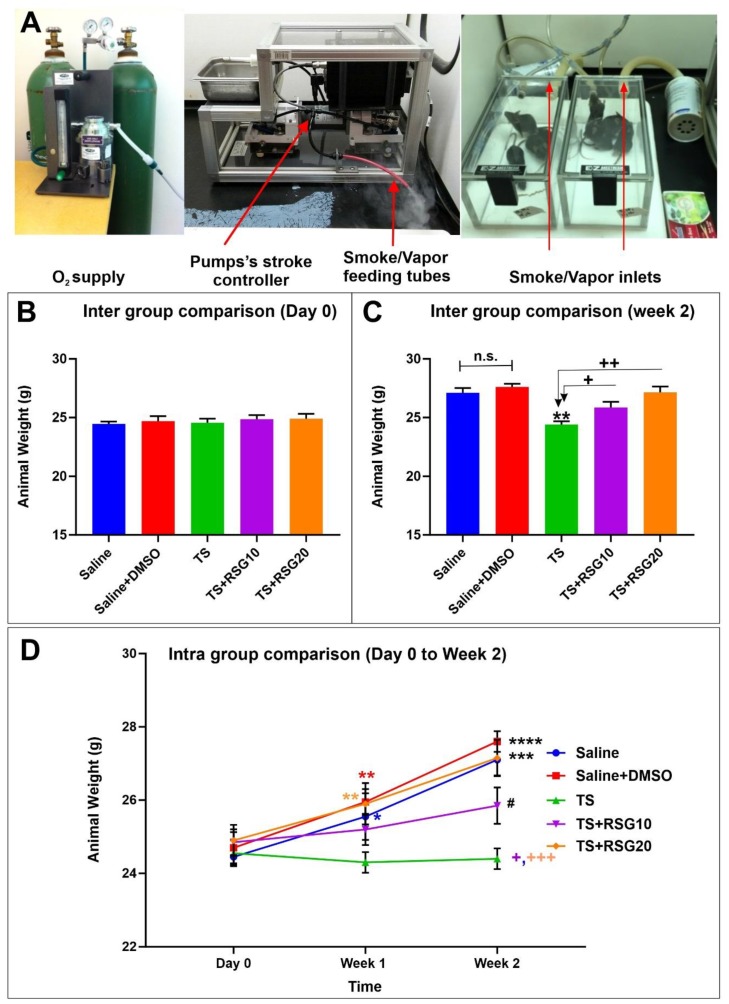
Effect of saline/DMSO/rosiglitazone (RSG) treatments with/without tobacco smoking’s (TS) exposure on body weight in vivo. (**A**) Set-up of the exposure of mice with chronic exposure to the TS. (**B**) Measurements of animals’ body weight did not show any significant difference between the tested groups at Day 0, however, at the end of the 2 weeks exposure (**C**), animal exposed to TS showed a significant lower body weight when compared to controls. The effect was abrogated by co-treatment with RSG. (**D**) Longitudinal assessment of animals’ body weight (all groups). Test mice also received a daily intraperitoneal injection of RSG. At the end of the experiment, brain tissue was collected, homogenized and processed for biochemical and molecular preparations. *n* = 4 biological replicates. *p < 0.05, **p < 0.01, ***p < 0.001 and ****p < 0.0001 versus saline. +*p* < 0.05, ++*p* < 0.01, +++*p* < 0.001 versus TS. #*p* < 0.05 TS + RSG 20 versus TS + RSG 10. n.s. = non statistical significance.

In-vivo studies are performed to evaluate and validate the protective effect of RSG against BBB damage and cerebrovascular dysfunctions caused by TS exposure. As shown in Figure 1A, TS generated by a CSM-SCSM cigarette smoking machine (CH Technologies, Westwood, NJ, USA) was forced directly into two airtight smoking chambers (Dimension- 24 L × 12 W × 12 H) housing the mice (4 mice/cage). The smoking inlet is dually connected to a feeding tube and a ventilator system supplying O_2_ (2 L/min) at atmospheric pressure (1 bar). During the interval between puffs, animals were receiving an uninterrupted supply of normal oxygenated air. Following the end of each smoking cycle, animals were transferred immediately back to their regular housings with food and water supply. Mice received a daily intraperitoneal injection of RSG before the first smoking cycle.

### 2.1. Decreased Harmful Effect of TS on Body Weight by RSG

Weight analysis was regularly performed to evaluate whether RSG dosing had any negative impact on body weight. As shown in Figure 1B,C we observed that there was a slight decrease in body weight in the group of untreated TS-exposed mice at the end of the 2 weeks of experimental testing. The effect of TS on body weight was reduced by the concomitant administration of RSG in a dose-dependent manner (see Figure 1C,D), demonstrating the lowered detrimental effect of TS by RSG, which accounts for the harmful effect of TS and the protective effect of RSG on the body weight.

### 2.2. Result for Nicotine and Cotinine Measurements

**Figure 2:**
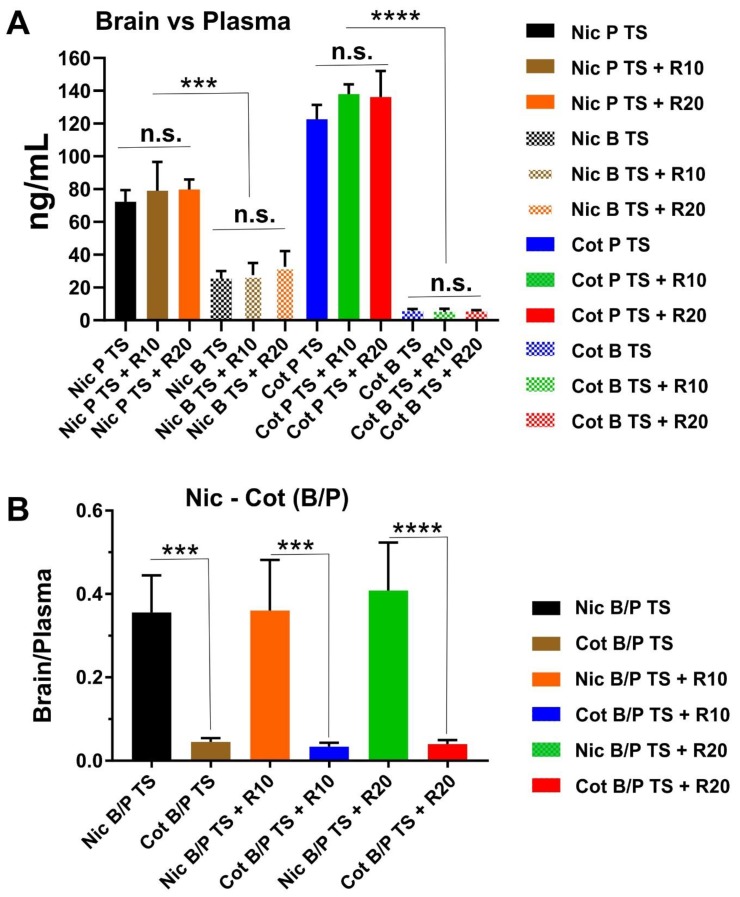
Plasma and brain levels of nicotine and cotinine in mice. (**A**) Side by side comparison of plasma “P” versus brain “B” levels, and (**B**) brain/plasma ratio of nicotine and cotinine across the main experimental groups, including TS exposed mice with and without RSG treatments. Note that nicotine and cotinine levels achieved across the various groups are not statistically different, thus indicating that levels of TS exposure achieved at the end of the 2 weeks among the test animals were very similar. Note also that differences in brain concentration between nicotine and cotinine, which correctly reflect the reduced blood–brain barrier (BBB) permeability to cotinine versus nicotine. *n* = 4 biological replicates. ***p < 0.001; ****p < 0.0001. n.s. = non statistical significance.

Plasma and brain levels of nicotine and cotinine in mice following two weeks of chronic exposure are shown in Figure 2A. Data showed that nicotine and cotinine concentrations both in the plasma and brain are comparable between the groups. This indicated that each group of animals was subjected to a very similar level of TS exposure. As previously reported, our exposure methods allow achieving the physiological concentration of nicotine and cotinine that are comparable to those observed in a heavy chronic smoker [28,29]. In Figure 2B we reported the calculated plasma to brain ratio of nicotine and cotinine. As expected, we did not observe any significant difference between the experimental groups. The data also reflected the poor brain permeability of cotinine when compared to nicotine.

### 2.3. Upregulation of PPARγ, NRF2—and Its Downstream Effectors NQO-1 and HO-1’s Expressions in a Dose-Dependent Manner

**Figure 3:**
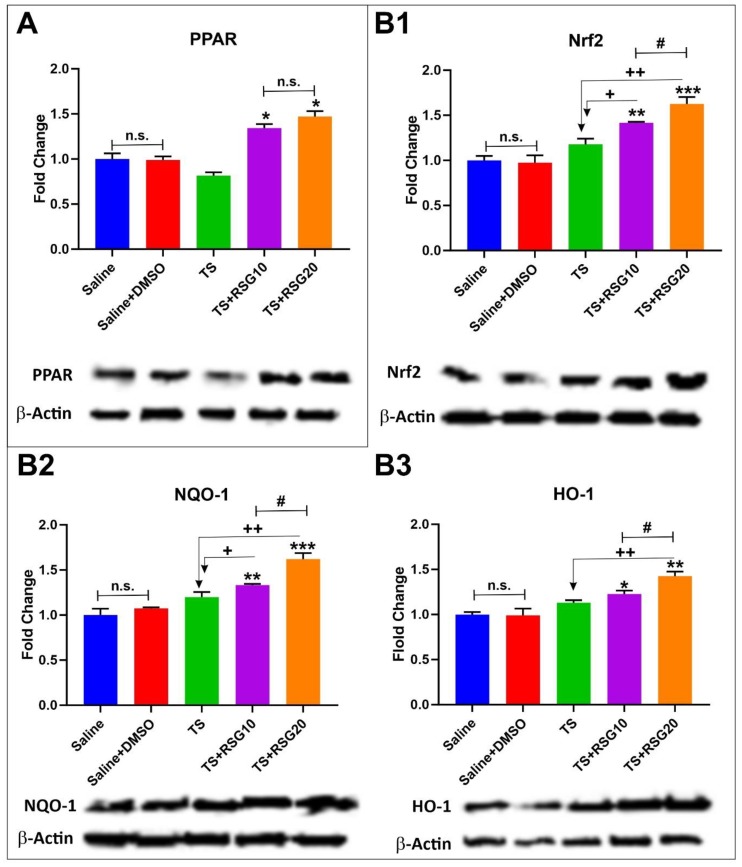
Dose-dependent protective effects of RSG against TS-induced oxidative stress. (**A**) Western blotting analysis emphasizing the effect of RSG on activation of the transcription factor peroxisome proliferator-activated receptor (PPARγ). (**B1**) Western-blot analysis emphasizing activation of the Nrf2 pathway. (**B2**,**B3**) Parallel to the increased Nrf2 expression levels by RSG, we also observe comparable upregulation of downstream detoxifying molecules NQO-1 and HO-1. *n* = 4 biological replicates. *p < 0.05, **p < 0.01, and ***p < 0.001 versus saline. +*p* < 0.05, ++*p* < 0.01, versus TS. **#***p* < 0.05 TS + RSG 20 versus TS + RSG 10. WB analyses report protein/β-actin ratios. n.s. = non statistical significance.

The effect of TS on the expression of Nrf2 and PPARγ was also evaluated, as demonstrated by western blot analysis in Figure 3. Treatment with RSG not only significantly stimulated the expression of PPARγ in a dose-dependent manner (Figure 3A), but equally enhanced that of Nrf2 (Figure 3B1). As shown in Figure 3B2,B3, increased expression of Nrf2 translated to similar upregulation of its downstream effectors NQO-1 and HO-1, as assessed by western blot analyses. That data demonstrated that RSG increased the overall activity of the Nrf2-ARE system in a dose-dependent manner.

### 2.4. RSG Decreases TS-Induced Loss of Blood–Brain Barrier Integrity

**Figure 4:**
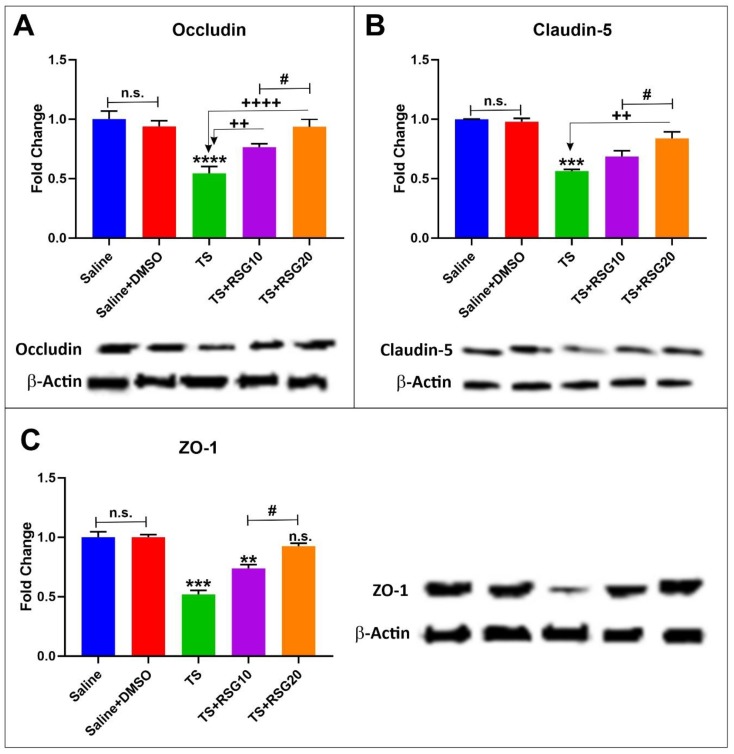
Dose-dependent protective effects of RSG against TS-induced loss of barrier integrity. Western blotting analysis demonstrating downregulation of TJ proteins ZO-1 (**A**), occludin (**B**) and claudin-5 (**C**) in animals exposed to TS. The effect was mitigated by RSG treatment in a dose-dependent manner. *n* = 4 biological replicates. *p < 0.05, **p < 0.01, ***p < 0.001 versus saline. +*p* < 0.05; ++*p* < 0.01 versus TS. #*p* < 0.05 TS + RSG 20 versus TS+RSG 10. WB analyses reported protein/β-actin ratios. n.s. = non statistical significance. *n* = 4 biological replicates.

Previous work by our group has shown that upregulated activity of the Nrf2 system is also accompanied by increased expression of tight junction proteins and decreased blood–brain barrier permeability both in vitro and in vivo [20,22,29,30]. Similarly, we assessed whether increased expression of Nrf2 by RSG also translated into upregulation of TJ protein expression using whole brain tissue homogenate. As demonstrated in Figure 4, chronic exposure to TS significantly downregulated the expression of Zonula occludens-1 (ZO-1); a TJ accessory protein (Figure 4C). Further, the expression level of main TJ proteins, including occludin and claudin-5 (Figure 4A,B) were also significantly downregulated when compared to controls. Notably, concurrent treatment with RSG counteracted the effect in a dose-dependent manner. TJ expression improvement by RSG over TS untreated mice was in strong accordance with that of Nrf2 as previously shown.

### 2.5. Decreased Pro-Inflammatory Effect of TS Exposure by RSG

**Figure 5:**
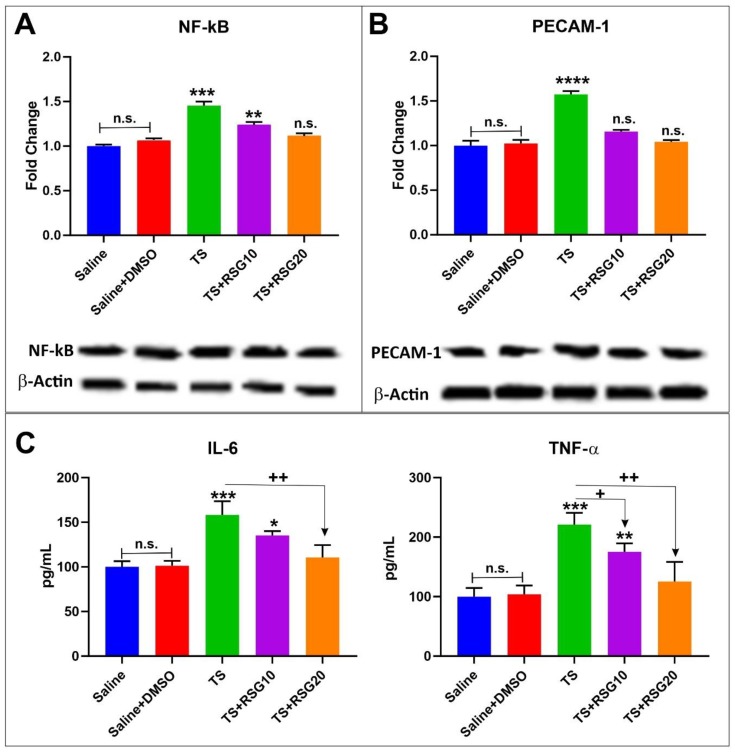
RSG decreases intracellular inflammation induced by TS. (**A**) Expression level of the inflammatory marker NF-kB, which was increased in cells treated with TS, but was downregulated by RSG. (**B**) Expression level of the inflammatory marker PECAM-1, which was increased in cells treated with TS, was downregulated by RSG. (**C**) ELISA of pro-inflammatory cytokines TNF-α and IL-6. RSG reduced TS induced TNF-α and IL-6 release. *n*= 4 biological replicates; *p < 0.05, **p < 0.01, ***p < 0.001, and ****p < 0.0001 versus saline. +*p* < 0.05, ++*p* < 0.01 versus TS. WB analyses report protein/β-actin ratios.

TS not only generates oxidative stress but also promotes inflammation linked to oxidative stimuli. Decreased and/or overwhelmed Nrf2 activity following chronic TS exposure become less efficient in contrasting oxidative stress stimuli, thus leading to increased inflammation. In this respect, RSG provided an effective countermeasure to the pro-inflammatory activity of TS. As shown in Figure 5A,B WB revealed a significant increase in the expression level of PECAM-1 and nuclear factor kappa-light chain-enhancer of activated B cells (NF-ĸB); a master regulator of inflammatory responses [31]. By contrast, RSG treatment decreased inflammation when compared to untreated TS-exposed animals. Specifically, our data show a reduction in the expression level of NF-ĸB (Figure 5A) and PECAM-1 (Figure 5B). The effect was also dose dependent. The analysis of pro-inflammatory cytokines by ELISA also revealed that RSG decreased TNF-α and IL-6 release in a dose-dependent manner in response to TS exposure (see Figure 5C).

## 3. Discussion

Oxidative stress, the redox imbalance caused by highly reactive oxygen species (ROS) which are either free oxygen radicals or reactive anions containing oxygen atoms, lead to cellular and tissue damage, such as lipoperoxidation of polyunsaturated fatty acids in membrane lipids, protein oxidation RNA oxidation, mitochondrial depolarization, DNA strand breakage and apoptosis. Additionally, ROS are crucial factors in the initiation and advancement of major cerebrovascular and neurodegenerative pathologies, including Alzheimer’s disease (AD), Parkinson’s disease (PD), amyotrophic lateral sclerosis (ALS), Huntington’s disease (HD), stroke and aging [22,31,32,33,34,35]. Oxidative stress plays an early, initiating role, as well as a potential late, by-product of neurodegeneration in these disease states [15]. Recent evidence has suggested that chronic exposure to TS is associated, in a dose-dependent manner, with dysfunction of normal endothelial physiology and subsequently in the pathogenesis of cerebrovascular disorders [1,6,9,10].

**Figure 6:**
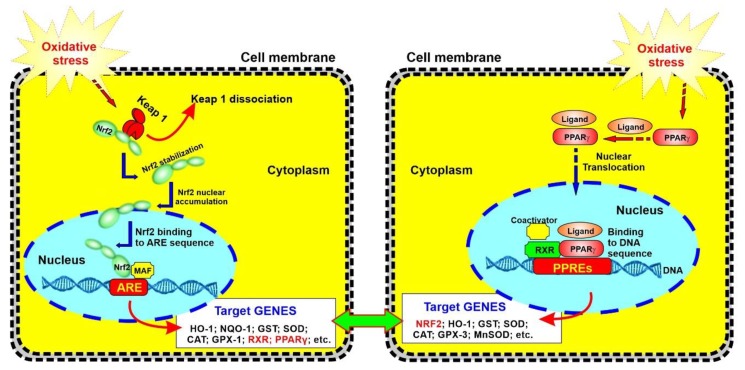
Schematic representation of crosstalk between Nrf2 and PPAR*γ* pathways in relation to OS and inflammation. Recent data by others suggests that Nrf2 and PPARγ might reciprocally reinforce the expression of one another, thus synergistically boost the antioxidative response system.

Nrf2, a basic region-leucine zipper (bZip) transcription Factor is the master regulator of multiple cytoprotective responses and a key regulator of redox homeostasis in cells [15,31]. Under basal conditions, Nrf2 is sequestered in the cytoplasm by its inhibitor, Kelch-like ECH-associated protein 1 (Keap1) [15]. In oxidative stress conditions, the cysteine residues of Keap1 become oxidized, releasing Nrf2, which is then free to translocate to the nucleus, which leads to it binding to the antioxidant response element (ARE) that is present in the regulatory regions of over 500 genes, allowing transcription of antioxidants [27,36]. Based on valid evidence, Nrf2 also enhances anti-inflammatory mediators, the activity of the proteasome and other transcription factors involved in mitochondrial biogenesis [37]. Recent studies from our group and other groups have highlighted the critical neuroprotective role of Nrf2 in a defense mechanism against oxidative stress, as well as regulation and maintenance of the BBB’s integrity and function [22,34,38,39,40]. Similarly to chronic smoking, an upregulation of Nrf2 diminishes the diabetic phenotype and the impairment in endothelial glucose uptake, causing the upregulation of tight junction protein expression and restoration of BBB integrity [41,42]. The results from our previous reports also suggested the pathological communalities between hyperglycemia and cigarette smoking at the BBB [21]. RSG, the PPARγ agonist and a member of the thiazolidinediones (TZDs) family has been currently assessed for the diseases associated with insulin resistance [27]. PPARγ is a member of a family of nuclear receptors that plays a pivotal role in regulating a huge number of genes implicated in glucose homeostasis and fatty acid metabolism. Jimenez et al. demonstrated the potentiality of PPARγ activation in attenuating high glucose-induced oxidative stress in endothelial cells and diabetic rats, associated with the involvement of Nrf2 [25]. In fact, they emphasized that PPARγ plays a vascular protective role against hyperglycemia-induced oxidative stress with the subsequent induction of HO-1 and upregulation of the Nrf2 [25]. Numerous studies confirmed that independent of their metabolic actions, RSG as a PPARγ agonist has a protective effect against oxidative stress caused by high glucose in diabetes and hypoperfusion [23,25,26,42,43,44]. As a member of the nuclear hormone receptor family, PPARγ is not only involved in adipogenesis and metabolic regulation, but also exerts pleiotropic anti-inflammatory effects, such as enhancing the transcription of anti-inflammatory and antioxidant genes, (several of which also up-regulated by Nrf2) [45]. In addition, PPARγ transrepresses key proinflammatory transcription factors, including NF-κB, STAT6 and AP-1 [45]. Recently, Cho et. al. demonstrated the presence of an inter-regulatory mechanism between PPARγ and Nrf2, which implies that their expression occurs in an interdependent and reciprocal manner. [46,47] (see also Figure 6). Moreover, the possible therapeutic use of this drug in the reduction of ROS and restoration of BBB integrity was investigated in our previous in vitro work.

In this preliminary study, we investigated the effect of RSG treatment on prevention and reduction of ROS-dependent BBB damage in response to chronic TS/EC exposure. In spite of the unknown mechanism of RSG, the previous study reported that RSG promotes endothelial cells’ protection through upregulation of Nrf2 by activating PPARγ, thereby protecting the blood–brain barrier against TS/EC induced dysfunction [2]. In line with these findings, in the present work, we evaluated the protective effect of RSG against tobacco smoke-dependent cerebrovascular toxicity using a rodent model of chronic smoking, previously developed and validated by our group [28,30]. Our results indicate that control mice receiving either saline or saline + DMSO followed a regular trend on the increase in body weight which accounts for lack of the harmful effect of DMSO as a solvent for RSG. It is noteworthy to point out that DMSO per se possesses anti-inflammatory activity and has been shown to repress inflammatory cytokine production [48] and promote hepatoprotection in acute treatment whereas if chronically administered may promote liver toxicity. In our case we did not observe any significant effect due to the relative low dosage used in our preparations.

The TS group demonstrated a loss of body weight when compared to control, which is also consistent with the well know metabolic stimulatory effect of TS. Loss of body weight was partially mitigated by RSG treatment in a dose-dependent manner, whereas RGS at the highest tested dose was not dissimilar from controls (Figure 1).

To confirm the in vitro protective effects of RSG, we assessed the impact of RSG on Nrf2 expression levels, as well as its downstream effector molecules NQO-1 and HO-1, which are known for exerting acute detoxification and cytoprotective functions. Despite a slight increase in Nrf2 level in untreated TS exposed mice receiving RSG, alongside TS showed improved Nrf2 expression/activity (Figure 3). Specifically, RSG-enhanced Nrf2 activation/expression was paralleled by a similar increase in the expression levels of NQO-1 and HO-1. The effect could both be due to a direct modulatory activity toward Nrf2 and/or PPARγ expression. Although it is not possible to dissect out the relative contribution of each target at this time, our data corroborate the findings of Jimenez et al. who demonstrates the upregulation of HO-1 (protective factor against vascular oxidative stress) in response to PPARγ activation [25]. Moreover, Cho et al. showed antioxidant effects of both Nrf2 and PPAR-*γ* and PPAR*γ* modulation by Nrf2, thus suggesting a positive role of PPAR-*γ* agonist in counteracting oxidative damage [37,47].

Oxidative Stress elicited by tobacco smoke triggers a pro-inflammatory response wherein leukocytes and monocytes are transported to the inflammation site and bind to the endothelial wall respectively. This has a cascading effect on the expression of selectins, pro-inflammatory and intercellular adhesion molecules [1]. Consequently, inhibitor of ĸB (IĸB) kinase complex is activated, which leads to their degradation and the subsequent release and translocation of NF-ĸB dimers to the nucleus, where it promotes transcription of genes responsible for the antioxidant response [31]. Prevention of TS-induced progressive upregulations of PECAM-1 and NF-kB were observed at the cerebrovascular level in TS exposed mice treated with RSG (Figure 5). These results are in line with the earlier reported in vivo work by Prasad et al., wherein an increase in systemic inflammation was observed upon chronic TS exposure in mice [29]. These also support our previous in vitro results wherein RSG reduced the expression of the PECAM-1 and NF-kB resulting from TS/EC exposure when compared to untreated TS/EC exposed cultures [2]. Existing evidence also suggests that TS exposure induces up-regulation and release of pro-inflammatory cytokines and decreases the release of cytokines could be indicative of reduced oxidative stress and inflammatory activity elicited by RSG (Figure 5).

Capillaries found in the central nervous system are different from those found in the rest of the body due to the BBB, which is a significant filter that protects the brain [49]. The BBB includes tight junction proteins, such as occludin and Claudins attaching together the cerebral endothelial cells, also scaffolding proteins, such as ZO-1, ZO-2 and ZO-3 anchoring the tight junction proteins in the endothelial cell [50]. The well-studied mechanisms for disruption of the BBB from oxidative stress are via matrix metalloproteinase (MMP) activation, NADPH oxidases and the toxicity of circulating free iron [50]. It is widely described that BBB integrity is deeply affected by oxidative stress, so that enhanced reactive oxygen species (ROS) generation leads to endothelium dysfunction and increased BBB permeability [51,52]. These alterations are mainly associated with the redistribution and/or altered expression of tight-junction proteins [16,51]. As demonstrated in Figure 4, down-regulation of ZO-1, claudin-5 and occludin were observed in response to TS exposure, thus confirming previous results [2,29,38]. Pre-treatment with RSG exhibited a protective effect against TS-induced loss of BBB TJs in a dose-dependent manner. The effect was not dissimilar to that of another oral antidiabetic drug, such as Metformin, which also exhibited the ability to upregulate Nrf2 expression/activity [29].

Taken together, these novel data clearly highlight that RSG is capable in preventing TS induced cerebrovascular dysfunction. Our results in the present work correlate very well with our previous in vitro study and strongly support previous observations. In the future, we plan to dissect out the mechanistic interrelationship between rosiglitazone, the PPARγ pathway and Nrf2 activity. Needless to mention that our experimental setting can only partially recapitulate the harmful effects produced by TS in chronic smokers over a period of years of decades. Ideally longer exposure periods could be used to reduce the gap to some extent. In addition, mice metabolism relative to nicotine conversion to cotinine is significantly faster than humans’. However, this difference was partially compensated by the exposure rate which allowed us to closely mimic the steady-state concentration of these compounds observed in heavy chronic smokers. One more limitation is the nature of this study that was aimed at validating our previous in vitro observations rather than dissecting out the specific mechanism of action. We are planning such a mechanistic driven study both in vitro and in vivo soon.

## 4. Materials and Methods

### 4.1. In Vivo Experimental Design

The animal study was conducted based on the animal protocol approved by the Institutional Animal Care and Use Committee, TTUHSC, Lubbock, Texas [29]. Twenty male C57BL/6J mice, (in the range 8–10 weeks old and body weight between 20 and 25 g) were purchased from Jackson Laboratory. After the animals arrived at the laboratory, they were given three days of recovery from the transport and for acclimatization in the new location. All animals were given unrestricted access to water and standard mouse chow. They were divided into five groups as shown in Table 1.

Test animals were chronically exposed (via direct inhalation) to side stream cigarette smoke (CS) derived from 3R4F research cigarettes (9.4 mg tar and 0.726 mg nicotine/cigarette) 6 times a day, 2 cigarettes/hour/8 animals, every day for 2 weeks. Cigarette exposure was set to meet the International Organization for Standardization/Federal Trade Commission (ISO/FTC) standard smoking protocol (35 mL puff volume, 2 s puff duration, 58 s intervals, 8 puffs per cigarette) [2,29]. CS was generated using a Single Cigarette Smoking Machines (SCSM, CH Technologies Inc., Westwood, NJ, USA) following previously published methods [6]. RSG was injected intraperitoneally and at the beginning of the day.

Two weeks of CS exposure and Drug injection was selected as the time course to reduce any possible toxicological effects caused by DMSO.

### 4.2. Materials and Reagents

Rosiglitazone (RSG # A00183, MW: 357.4) was obtained from the Adipogen. Reagents and chemicals were purchased from Sigma-Aldrich (St. Louis, MO, USA) or Bio-Rad Laboratories (Hercules, CA, USA). The antibodies used in this study were purchased from the various sources: Rabbit anti-ZO-1 (#402200), mouse anti-occludin (#331500) and anti-claudin-5 (#4C3C2) from Life Technologies; rabbit anti-PPARγ (#331500) from Invitrogen; rabbit anti-Nrf2 (#NBP-1-32822) from Novus Biologicals. Mouse anti-PECAM-1 (#sc-376764), mouse anti-NQO-1 (#sc-376023), mouse anti-HO1 (#sc-390991) and mouse anti-NFκB-p65 (#sc-(F-6)-8008) from Santa Cruz Biotechnology. Donkey anti-rabbit (#NA934) and sheep anti-mouse (#NA931) HRP-linked secondary antibodies were obtained from GE Healthcare (Piscataway, NJ, USA).

### 4.3. Drug Administration

RSG was dissolved in DMSO/sterile saline (1:10) and administered daily via intraperitoneal injections of dose levels of 10 or 20 mg/kg, with dose volumes of 20 mL/kg to mice either exposed to TS (mixed with oxygenated air) or on oxygenated air alone (controls) for 2 weeks, as mentioned earlier. An equal volume of DMSO/saline (1:10) was used for the control group which received either oxygenated air or TS [26,53].

### 4.4. Tissue Preparation

Mice were sacrificed one day after the last day of TS exposure cycle to collect their brains for subsequent biochemical and molecular analysis. Briefly, a cut was made at the nape and extended along the midline from the dorsal cervical area to the tip of the nose. After pulling the skin away from the skull laterally, a cut through the spine at the base of the skull was made using a dedicated pair of sterile brain harvest scissors. The skull was opened by placing the point of the scissors in the foramen magnum and cutting along the midline. The parietal bones were levered away from the brain through the flat end of the scissor blade. The nerve attachments at the brain stem and the optic chiasm beneath the brain were disrupted using the closed point of thumb forceps. The brain was then dropped from the skull into sterile medium [29].

### 4.5. Preparation of Protein Extracts and Western Blotting

To harvest the proteins, cells and homogenized brain tissues were lysed using either subcellular protein fractionation kit for cultured cells (Thermo scientific, Waltham, MA, US; cat#78840) or RIPA lysis buffer, so that total nuclear, cytosolic and membrane fractions were collected by centrifugation at 14,000 *g* for 30 min. Samples were then aliquoted and stored at −80 °C for the next protein expression analysis by western blotting. Proteins’ quantification was carried out using Pierce BCA Protein Assay Kit (Thermo Scientific, cat# 23225). Samples (60–90 μg for tissue lysates and) were then prepared, as described in our previous lab report [2,20,29]. Briefly, denatured samples were run on SDS-PAGE (4%–15% gradient gel) and transferred to PVDF or nitrocellulose membranes for further blotting. The membranes were washed with Tris-buffered Saline (TBS) (10 mmol/l Tris-HCl, pH 7.4, 150 mmol/L NaCl) containing 0.1% Tween-20 (Tween-TBS), blocked for 1 h with Tween-TBS containing 5% non-fat dry milk, and incubated with primary antibodies prepared in Tween-TBS containing 5% bovine serum albumin (BSA) overnight at 4 °C. The following day, for immunodetection, cells were washed and then incubated with the secondary antibody prepared in Tween-TBS containing 5% BSA for 2h. The protein Band densities were analyzed by Image Studio Lite Ver 3.1 and calculated as fold change/percentage change over control protein expression. All protein quantifications were adjusted for the corresponding β-actin level, which was not consistently changed by the different treatment conditions.

### 4.6. ELISA

Tissue lysates from mice were analyzed by Quantikine ELISA kits (R & D systems, Minneapolis, MN, USA) for the quantitative determination of TNF-α and IL-6 in accordance to the manufacturer’s protocol.

### 4.7. Nicotine and Cotinine Measurements in Brain and Plasma.

Nicotine and cotinine concentrations in plasma and brain tissue were assessed previously described by our group [28]. Once the last smoking cycle in last day was completed, we collected (within 30 min) a 100 µL blood sample by cardiac puncture. The sample was centrifuged at 1300 g for 10 min to obtain the plasma, which was stored at −80 °C. Following decapitation, the brain was removed and divided into 2 equal samples. One part was stored at −80 °C for further molecular and biochemical analyses; the other part was instead homogenized in water (1:10 ratio) and used immediately for cotinine and nicotine quantification using a rapid, sensitive and very specific UHPLC-MS/MS method developed and validated by our group [28]. This method allows for simultaneous quantification of nicotine and cotinine in mouse plasma and brain homogenates.

### 4.8. Statistical Analysis

Data from all experiments were expressed as mean ± standard deviation (SD). Sample size was chosen based on previously published manuscript by us and others to produce 80% power and a type 1 error rate = 0.05. Blind analysis was performed by one-way ANOVA using GraphPad Prism 6 Software Inc. (La Jolla, CA, USA). Post multiple comparison tests were performed as with Tukey’s or Dunnett’s test as suggested by the software. *P* values < 0.05 were considered statistically significant.

## 5. Conclusions

In summary, at the cerebrovascular level, cigarette smoking can cause oxidative damage, trigger a strong inflammatory cascade and severely impair endothelial physiology, thus leading to the onset and/or progression of several major cerebrovascular disorders. In this study, the protective effect of RSG against TS-induced damages was investigated in a rodent model with the scope to validate previous in vitro results [2]. The key role of Nrf2 in maintaining BBB functional integrity and endothelial structure was indirectly confirmed, thus supporting previous in vitro and in vivo evidences. In the current experimental setting, we proved that RSG can effectively counteract previously observed TS-dependent impairments of the BBB, including the loss of BBB integrity, OS damage and inflammation. RSG’s protective mechanism seems to depend upon Nrf2 and PPARγ expression. Studies by others have shown that there is a crosstalk and loop regulatory pathways between those two factors that acts on repressing inflammation while promoting the activation of the antioxidative response system [45,46,47]. Although outside the scope of this work, we plan for future experiments, to dissect out the Nrf2-PPARγ contribution to RSG’s protective effects against TS and better understand the details of the crosstalk between those two factors with the use of Nrf2-KO mice model and selective silencing.

In summary, our data suggest that RSG could have promising therapeutic potential to prevent cigarette-induced cerebrovascular dysfunction, and possibly other xenobiotic substances which may impact the BBB via oxidative-stress-mediated effects.

## Figures and Tables

**Table 1:** Experimental design.

	Group 1	Group 2	Group 3	Group 4	Group 5
Saline	✓	-	-	-	-
Saline + DMSO	-	✓	✓	✓	✓
RSG 10 mg/kg	-	-	-	✓	-
RSG 20 mg/kg	-	-	-	-	✓
TS exposure	-	-	✓	✓	✓

## Abbreviations

ARE	Antioxidant Response Element
BBB	Blood–brain barrier
CNS	Central nervous system
CS	Cigarette Smoke
2DM	Type 2 Diabetes Mellitus
EC	Electronic Cigarette
FTC	Federal Trade Control
HG	Hyperglycemia
HO-1	Heme Oxygenase 1
ISO	International Organization for Standardization
Keap1	Kelch-like ECH-associated protein 1
NF-ĸB	Nuclear factor kappa-light chain-enhancer of activated B cells
NQO-1	NAD(P)H: Quinone reductase I
Nrf2	Nuclear factor erythroid 2-related factor
OS	Oxidative stress
PPARγ	Peroxisome proliferator-activated receptor gamma
PECAM-1	Platelet Endothelial Cell Adhesion Molecule-1
ROS	Reactive Oxygen Species
RSG	Rosiglitazone
SCSM	Single Cigarette Smoking Machine
TJ	Tight Junction
TS	Tobacco smoke
TZD	Thiazolidinediones
ZO-1	Zonula occludens-1
